# Deer Antler S‐Adenosylmethionine Ameliorates Depression‐Like Behaviors and Neuroinflammation in CUMS Mice

**DOI:** 10.1111/cns.70569

**Published:** 2025-09-10

**Authors:** Yongjian Liu, Chenyang Han, Li Guo, Wenyan Li, Shasha wu, Jian Sheng, Liping Zhai, Heping Shen, Genghuan Wang

**Affiliations:** ^1^ Interventional Department First Affiliated Hospital of Dalian Medical University Dalian China; ^2^ The Second Affiliated Hospital of Jiaxing University Jiaxing Zhejiang China

**Keywords:** CUMS, depression, glycolysis, microglia, S‐adenosylmethionine

## Abstract

**Aim:**

To investigate the effects and mechanisms of S‐adenosylmethionine (SAM) from deer antler on improving depression‐like behaviors in chronic unpredictable mild stress (CUMS) mice.

**Methods:**

The CUMS method was used to establish a mouse depression model. The relationship between SAM and HIF‐1α was analyzed by small molecule‐protein docking and molecular dynamics simulation.

**Results:**

Network pharmacology analysis showed that the effect of SAM is related to HIF‐1α. When shRNA‐HIF‐1α was used to silence the expression of HIF‐1α, the effect of SAM was inhibited. The improvement of depression‐like behaviors in mice was not significant, and inflammatory factors were no longer further downregulated.

**Conclusion:**

SAM can improve depression‐like behaviors in CUMS mice and enhance their mobility. SAM inhibits neuroinflammation and glycolysis, and also suppresses the activation of microglia.

## Graphical abstract

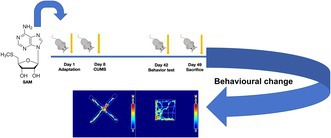

## 1 Background

Microglia are immune cells in the brain, accounting for about 10% of brain cells, responsible for protecting and maintaining brain activity [1]. Microglia have high metabolic flexibility and can adjust their energy metabolism pathways according to environmental changes [2]. Under homeostatic conditions, microglia mainly rely on glucose oxidative phosphorylation to produce energy [3]. However, under pathological conditions, such as depression, microglia may switch to glycolysis to produce energy [4, 5]. This metabolic shift has an important impact on the function of microglia and the pathogenesis of depression. In depression, microglia are activated and release pro‐inflammatory cytokines. This activated state of microglia tends to use glycolysis to produce energy to support its inflammatory response. Although glycolysis is less efficient, it can quickly provide energy to support the rapid response and inflammatory reaction of microglia [6, 7]. Studies have shown that in various stress‐induced depression animal models, the glycolysis pathway of microglia is activated, accompanied by the appearance of depression‐like behaviors. Therefore, improving the level of glycolysis in microglia can be a new means of treating depression‐related neuroinflammation [8].

Deer antler refers to the young horn of a male deer that has not yet ossified, rich in various amino acids, trace elements, neurotransmitters, and other active ingredients. Traditional Chinese medicine believes that deer antler has the effects of warming and tonifying the kidney and nourishing blood and energy, and is widely used in the treatment of neurasthenia, depression, and other symptoms [9]. Studies have found that many active ingredients in deer antler have regulatory effects on the central nervous system, which can improve the imbalance of neurotransmitters and thus alleviate depressive symptoms. The active ingredients in deer antler extracts, such as peptides and polysaccharides, can act on the neurotransmitter system, regulate the content and function of neurotransmitters such as serotonin and dopamine, and thus achieve an antidepressant effect [10]. S‐adenosylmethionine is a new active substance in deer antler, and there is no clear report on its antidepressant effect and mechanism. We further analyze the effect and mechanism of S‐adenosylmethionine in deer antler from the perspective of glycolysis.

## 2 Material and Methods

### 2.1 Main Materials and Reagents

S‐adenosylmethionine from deer antler (SAM, gifted by Dongfeng Sika Deer Comprehensive Experimental Station of Jilin Agricultural University), C57BL/6J mice (purchased and raised by Nanjing Model Animal Research Institute), ELISA kits for IL‐1β, IL‐6, TNF‐α (Nanjing Jiancheng Bioengineering Institute, NO.H002, H007, H052, H015), AAV‐CMV‐shRNA‐HIF‐1α (provided by ViGene), tissue fluorescence staining kit (Solarbio, NO.CA1142), monoclonal antibody IBA‐1 for tissue fluorescence staining (Abcam, NO.ab178846), monoclonal antibodies HIF‐1α, HK2, PK (Abcam, NO.ab179483, ab227198, ab300389), elevated plus maze and animal movement trajectory tracking system (EthoVision XT) (Noldus), open field test box (XR‐XZ301) (Noldus). Primary microglia (provided by Procell Biotechnology Co. Ltd.), pyruvate and lactate detection kits (Nanjing Jiancheng Bioengineering Institute, NO.A09, A081).

### 2.2 Animal Model

Chronic Unpredictable Mild Stress (CUMS) [11]: The modeling time of CUMS was 5 weeks. The mice were adaptively fed for 1 week. During the CUMS modeling period, the mice were given one chronic unpredictable mild stress stimulus every day, mainly including (a) camphor ball intervention for 24 h, odor interference. (b) 2 h of restraint for mice. (c) 3 h of tail clamping for mice. (d) living in a humid environment for 24 h. (e) shaking the mouse cage for 5 min. (f) tilting the mouse cage for 24 h. (g) swimming in ice water for 5 min. (h) 24 h of light–dark reversal. (i) 24 h of water deprivation. (j) 24 h of food deprivation. The time and order of each stimulus given to the mice were random, with no repetition within 3 days, following the principles of unpredictability and randomness.

#### 2.2.1 Experiment 1

We used C57BL/6J mice, divided into Control, CUMS, SAM‐L, and SAM‐H groups, with 10 mice in each group. The Control group was the control mice, and the other four groups were treated according to the CUMS model construction method. SAM‐L and SAM‐H were given 10 mg/kg and 20 mg/kg of deer antler S‐adenosylmethionine daily, respectively, with a gavage once a day, consistent with the CUMS cycle.

#### 2.2.2 Experiment 2

Using 57BL/6 J mice, AAV‐CMV‐shRNA‐HIF‐1α was injected into the brain stereotactically at a dose of 9 × 10^13^ vg/mL, 100 nL, 10 nL/min to silence HIF‐1α. The HIF‐1α‐KO mice were divided into Control, CUMS, and SAM groups. The CUMS construction method was consistent with the above. The SAM group used 20 mg/kg of deer antler S‐adenosylmethionine, with a gavage once a day.

### 2.3 Sucrose Preference Test (SPT) [12]

After 1 week of adaptive feeding of mice, the SPT experiment lasted for 4 days. On the first day, a 2% sucrose solution bottle was tied to the mouse cage for 24 h. On the second day, two bottles were placed at the same height, one with 2% sucrose solution and the other with distilled water. After 12 h, the positions of the bottles were swapped. On the third day, water and food were deprived. On the fourth day, 2% sucrose solution and distilled water were placed at the same height for 3 h, with the positions swapped after 1.5 h. At the end of the test, the consumption of 2% sucrose solution and distilled water by the mice was measured. Sucrose preference (%) = sucrose solution consumption/(sucrose solution consumption + distilled water consumption) × 100%.

### 2.4 Tail Suspension Test (TST) [13]

The tip of the mouse's tail (1 cm) was fixed with tape, and the mouse was suspended upside down from a hanging rod with its tail 20 cm from the ground. The experiment lasted for 6 min, and the time the mouse was immobile within 4 min was recorded. Under these conditions, the mouse would struggle and swing but would eventually become motionless after failing to escape, a state known as behavioral despair. The time the mouse was suspended and motionless was recorded.

### 2.5 Swimming Test (FST) [14]

A glass cylinder with a diameter of 12 cm and a height of 20 cm was filled with warm water (25°C). The mice were allowed to adapt to swimming in the pool for 2 min, after which the immobile time of the mice during swimming was recorded. The criterion for immobility was that the mouse's limbs were motionless and floating on the surface of the water, with only the head breathing and no obvious struggling. The immobile time of the mice was recorded.

### 2.6 Elevated Plus Maze (EPM) [15]

The elevated plus maze is a maze with four choices. Two directions are open spaces without walls, known as open arms. The other two are closed arms. The mouse maze was 40 cm from the ground, with arms 30 cm long and 5 cm wide. The mouse was placed on the platform facing the open arm. The movement trajectory of the mouse in the elevated plus maze for 5 min was recorded using the behavioral testing software SMART, and the time spent in the open arms and the number of entries into the open arms were analyzed.

### 2.7 Open Field Test (OFT) [16]

One hour before the test, the mice were placed in the center area of the open field and allowed to move freely for 10 min to familiarize themselves with the environment. The software recorded the behavior and corresponding time of the mice in real time. The mice were then removed from the open field box and returned to their cages. Before testing the next mouse, the entire open field area was cleaned with 750 mL/L ethanol and paper towels. The total movement distance, average movement speed, time spent in the central area, and active time of the mice were recorded.

### 2.8 Elisa

We detected the levels of inflammatory factors IL‐6, IL‐1β, TNF‐α in the mouse cortex, and the levels of these factors in the culture medium after centrifugation in vitro experiments. The tissues were ground in liquid nitrogen, lysed with NP‐40 to obtain the supernatant, and the culture medium was centrifuged to obtain the supernatant. The ELISA kit instructions were followed for detection, and the results were expressed as pg/mg prot.

### 2.9 Lactate and Pyruvate Detection

Lactate and pyruvate detection kits were purchased from Nanjing Jiancheng Bioengineering Institute. Lactate detection was performed using the standard curve method. Briefly, the enzyme reserve solution and diluent were mixed at a ratio of 1:100, and the coloring solution was diluted to 6 mL for later use. The standards were diluted, and the enzyme working solution and coloring agent were added and incubated in a cell culture incubator for 10 min. The absorbance at 530 nm was measured to plot the standard curve. Following the ELISA method, brain tissue supernatant was prepared to measure lactate levels. Pyruvate detection was also performed using the standard curve method, with the standard curve plotted according to the instructions, and the absorbance at 505 nm measured using a microplate reader to determine the pyruvate levels in brain tissue supernatant.

### 2.10 Tissue Fluorescence Staining

Brain tissue was sectioned at a thickness of 4 μm using a Leica CM1500 cryostat and mounted on slides. The tissue slides were fixed with 40 g/L paraformaldehyde at room temperature for 15 min, washed three times with PBS for 5 min each, and blocked with 100 mL/L horse serum for 1 h. The IBA‐1 monoclonal antibody was added to the sections and incubated overnight at 4°C. The primary antibody was washed with PBS three times for 5 min each, and then the secondary antibody was added and incubated at room temperature in the dark for 2 h. The slides were washed with PBS three times for 5 min each, and then Hoechst dilution solution (1:5000) was added and incubated at room temperature for 15 min. The slides were mounted with 750 g/L glycerol and stored in a −20°C refrigerator in the dark after mounting.

### 2.11 Western‐Blotting

Proteins were extracted from cells by NP‐40 lysis, and protein concentration was determined and adjusted using the BCA method. Protein electrophoresis was performed using precast gels. The protein solution was supplemented with 5× loading buffer to a volume of 20 μL, boiled for 8 min, and then electrophoresed at 80 V. The voltage was then increased to 120 V to continue electrophoresis, and the protein was transferred to a membrane at a constant current of 300 mA for 0.5–2 h. The membrane was blocked with 5% skim milk for 2 h, and the primary antibody was diluted with TBST and incubated overnight on a rocker. The secondary antibody, conjugated with horseradish peroxidase, was then incubated. After incubation, chemiluminescence was used for detection, and Image Pro‐Plus 6.0 software was used for densitometric analysis.

### 2.12 Statistical Methods

All measurement data were expressed as (*n* ± SD). The data were analyzed and processed using SPSS 17.0 software. After the homogeneity of variance test, two independent sample *t*‐tests were used for comparisons between two groups; one‐way ANOVA was used for comparisons among three or more groups, followed by the LSD method for pairwise comparisons. All tests were two‐sided, and *p* < 0.05 was considered statistically significant.

## 3 Results

### 3.1 S‐Adenosylmethionine Improves Depression‐Like Behaviors and Neuroinflammation in CUMS Mice

**FIGURE 1:**
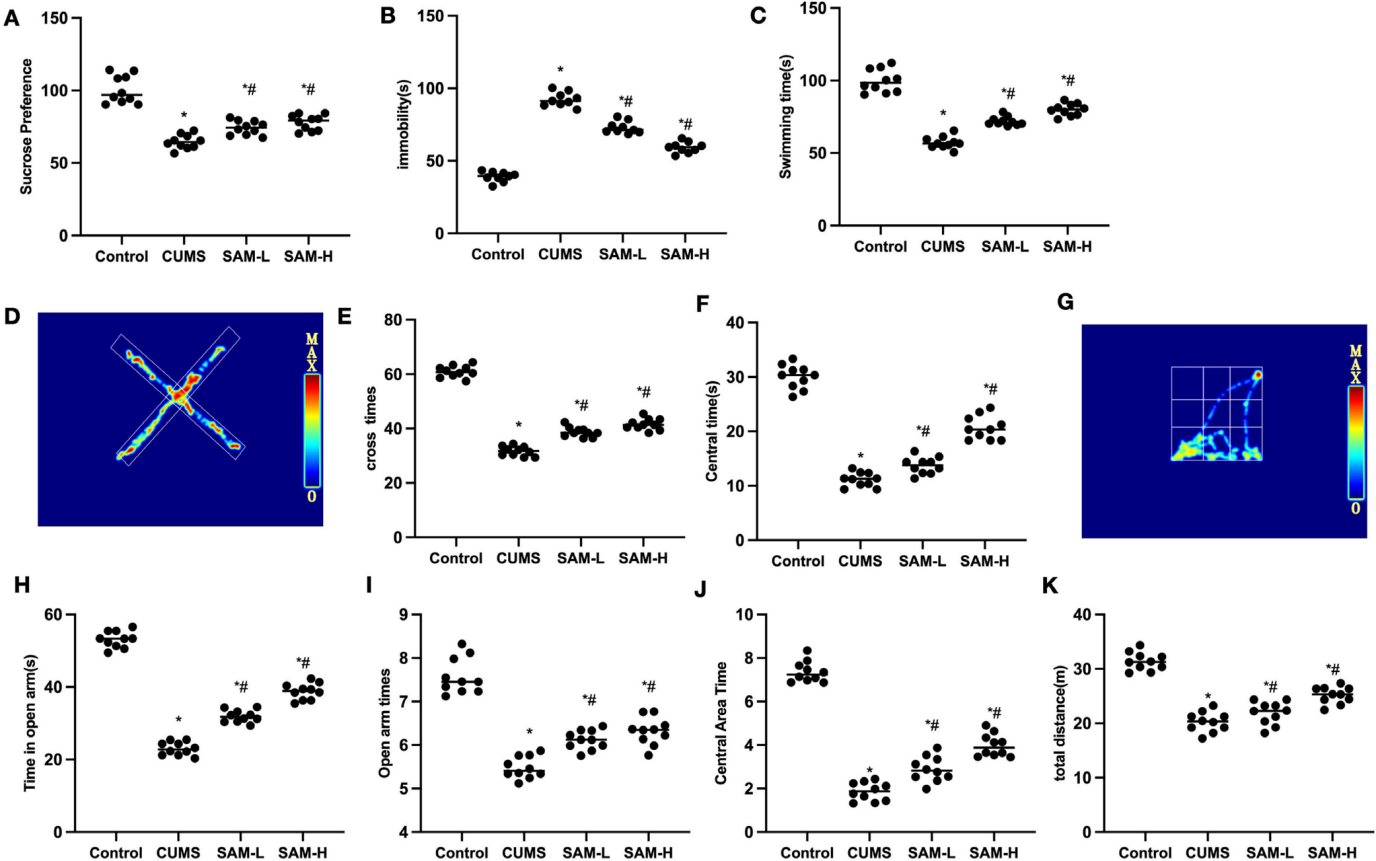
The effect of SAM on depression‐like behaviors in CUMS mice. (A) SPT (*n* = 10), the sucrose preference in CUMS mice was decreased, and SAM can increase the preference, significantly higher than CUMS. (B) TST (*n* = 10), the hanging time in CUMS mice was longer than that in the Control group; SAM can reduce the hanging time, with a significant difference compared to CUMS. (C) FST (*n* = 10), the swimming time in CUMS mice was shorter than that in the Control group; SAM can increase the swimming time. (D–F) EPM (*n* = 10); both the time spent in the open arms and the number of entries into the open arms were lower in CUMS mice than in the Control group, and SAM can increase these parameters. (G–K) OFT (*n* = 10), the total movement distance, average movement speed, time spent in the central area, and active time were all lower in CUMS mice than in the Control group; SAM can increase the movement distance, movement speed, and active time of the mice. Compared with the Control group, *p < 0.05; compared with the CUMS group, #*p* < 0.05.

CUMS mice exhibited obvious depression‐like behaviors, with decreased motor and cognitive abilities, increased glycolysis levels, and upregulated activation of microglia. SAM can improve depression‐like behaviors in mice, inhibit glycolysis levels, and reduce the activation of microglia. The SPT results showed that the sucrose preference in CUMS mice was decreased, and SAM can increase the preference, significantly higher than CUMS (Figure 1A). The TST results showed that the hanging time in CUMS mice was longer than that in the Control group, and SAM can reduce the hanging time, with a significant difference compared to CUMS (Figure 1B). The FST results showed that the swimming time in CUMS mice was shorter than that in the Control group, and SAM can increase the swimming time (Figure 1C). The EPM results showed that both the time spent in the open arms and the number of entries into the open arms were lower in CUMS mice than in the Control group, and SAM can increase these parameters (Figure 1D–F). The OFT results showed that the total movement distance, average movement speed, time spent in the central area, and active time were all lower in CUMS mice than in the Control group, and SAM can increase the movement distance, movement speed, and active time of the mice (Figure 1G–K).

**FIGURE 2:**
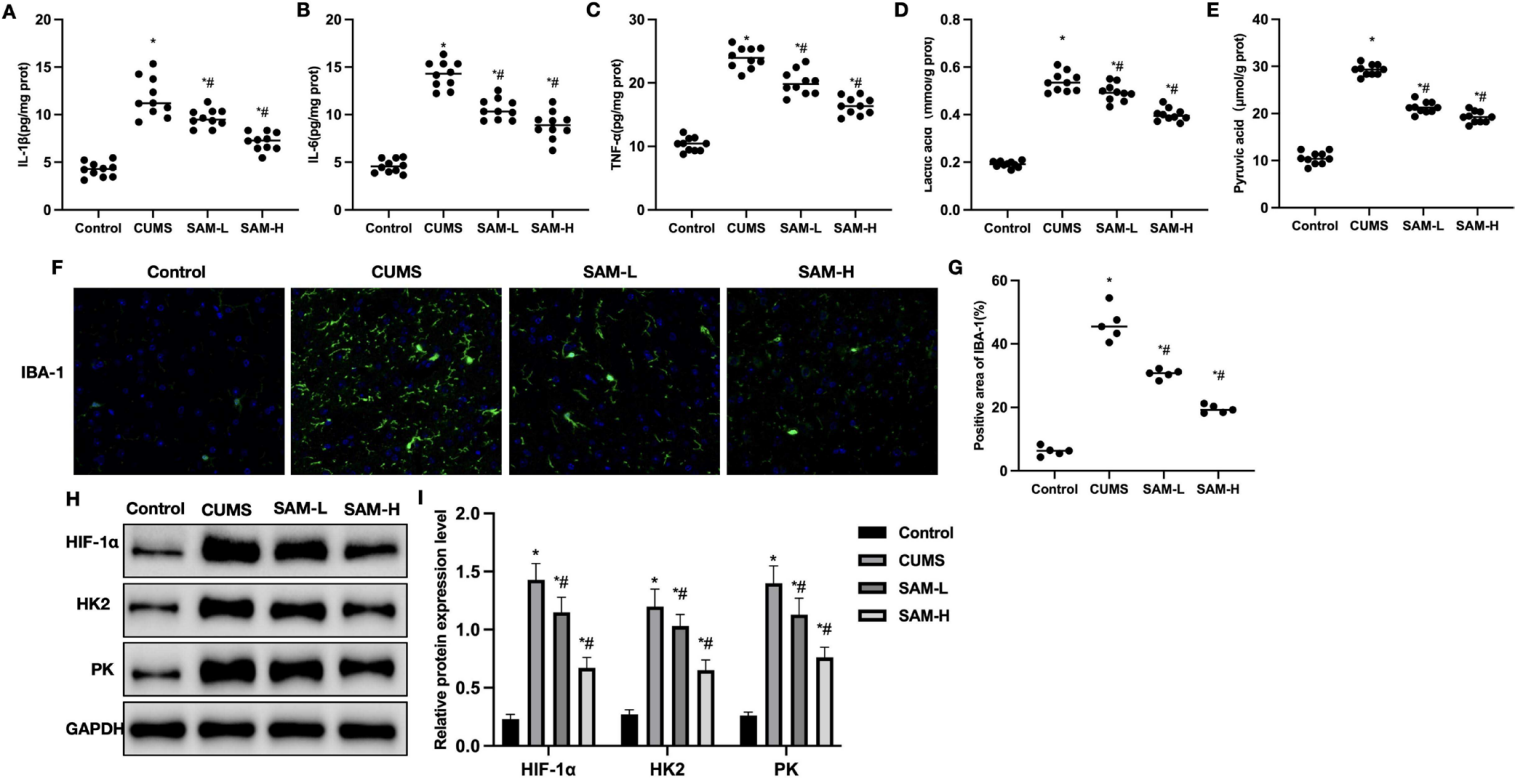
The effect of SAM on pathology in CUMS mice. (A–C) ELISA (*n* = 10); the levels of inflammatory factors in the cortex of CUMS mice were higher than those in the Control group, and SAM can reduce the expression of these factors. (D–E) Lactate and pyruvate detection (*n* = 10); the levels of pyruvate and lactate in brain tissue of CUMS mice were significantly higher than those in the Control group, and SAM can reduce the levels of pyruvate and lactate in the tissue. (F, G) IBA‐1 staining (*n* = 5); the number of IBA‐1 positive cells was significantly higher in CUMS mice than in the Control group, and SAM can reduce the expression of IBA‐1 and the number of IBA‐1 positive cells. (H, I) Relative protein expression levels (*n* = 5); the levels of HIF‐1α, HK2, and PK were significantly higher in CUMS mice than in the Control group, and SAM can significantly reduce the protein levels, inhibiting the expression of HIF‐1α, HK2, and PK. Compared with the Control group, *p < 0.05; compared with the CUMS group, #*p* < 0.05.

Inflammatory factor detection showed that the levels of inflammatory factors in the cortex of CUMS mice were higher than those in the Control group; SAM can reduce the expression of these factors (Figure 2A–C). Pyruvate and lactate detection showed that the levels of pyruvate and lactate in brain tissue of CUMS mice were significantly higher than those in the Control group, and SAM can reduce the levels of pyruvate and lactate in the tissue (Figure 2D,E). IBA‐1 staining showed that the number of IBA‐1 positive cells was significantly higher in CUMS mice than in the Control group, and SAM can reduce the expression of IBA‐1 and the number of IBA‐1 positive cells (Figure 2F,G). The relative protein expression levels showed that the levels of HIF‐1α, HK2, and PK were significantly higher in CUMS mice than in the Control group, and SAM can significantly reduce the protein levels, inhibiting the expression of HIF‐1α, HK2, and PK (Figure 2H,I).

### 3.2 Network Pharmacology Analysis of SAM‐Glycolysis‐Inflammatory Response

**FIGURE 3:**
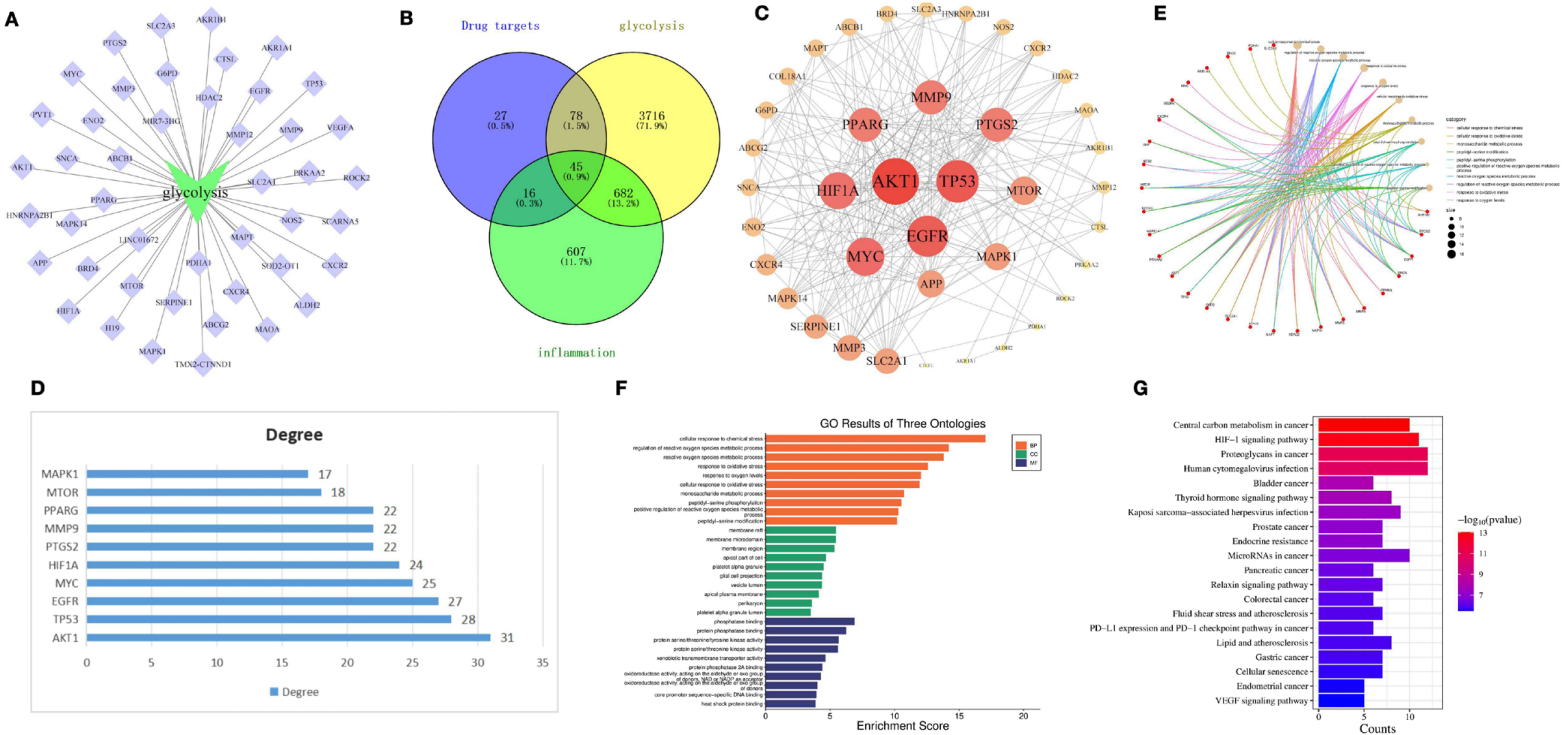
Results of network pharmacology analysis of SAM. (A) Display of targets related to glycolysis. (B) A total of 45 targets were shown after the intersection of SAM‐glycolysis‐inflammation response. (C, D) Display and ranking of targets. (E, F) Results of GO analysis. (G) Results of KEGG analysis.

We analyzed the targets related to glycolysis (Figure 3A). After intersecting SAM‐glycolysis‐inflammatory response, a total of 45 targets were shown (Figure 3B). We performed relevance analysis on the targets and displayed the top 10 targets (Figure 3C,D). GO analysis showed that SAM is associated with 25 biological information pathways (Figure 3E,F). KEGG analysis results showed that HIF‐1α‐mediated signaling is closely related to SAM (Figure 3G).

### 3.3 Effect of SAM Suppressed by HIF‐1α‐KO

**FIGURE 4:**
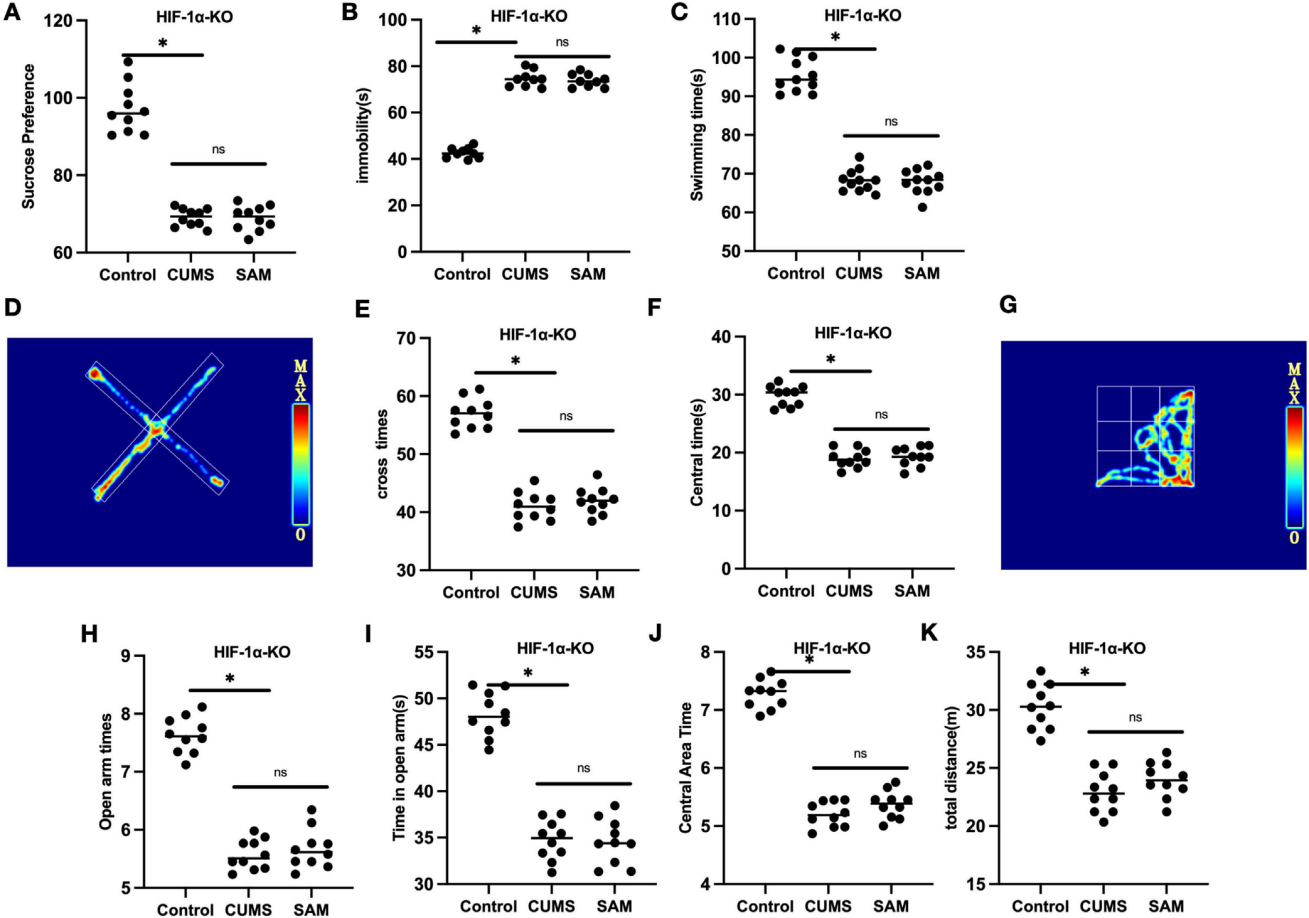
The effect of SAM on depression‐like behaviors in CUMS mice after HIF‐1α‐KO. (A) SPT (*n* = 10), no difference in sucrose preference between CUMS and SAM groups. (B) TST (*n* = 10); no significant difference in hanging time between CUMS and SAM groups. (C) FST (*n* = 10), no significant difference in swimming time between CUMS and SAM mice. (E, F) EPM (*n* = 10); no significant difference in the time spent in the open arms and the number of entries into the open arms between CUMS and SAM groups. (G–K) OFT (*n* = 10), no significant difference in the total movement distance, average movement speed, time spent in the central area, and active time between CUMS and SAM groups. Group comparison, *p < 0.05; ns, *p* > 0.05.

After silencing HIF‐1α with shRNA‐HIF‐1α, HIF‐1α could not be further expressed, and SAM could not further improve the depression‐like behavioral changes, nor did it have a significant effect on neuroinflammation and the activation of microglia. The SPT results showed no difference in sucrose preference between CUMS and SAM groups (Figure 4A). The TST results also showed no significant difference in hanging time between CUMS and SAM groups (Figure 4B). The FST results showed no significant difference in swimming time between CUMS and SAM mice (Figure 4C). The EPM results showed no significant difference in the time spent in the open arms and the number of entries into the open arms between CUMS and SAM groups (Figure 4D–F). The OFT results showed no significant difference in the total movement distance, average movement speed, time spent in the central area, and active time between CUMS and SAM groups (Figure 4G–K).

**FIGURE 5:**
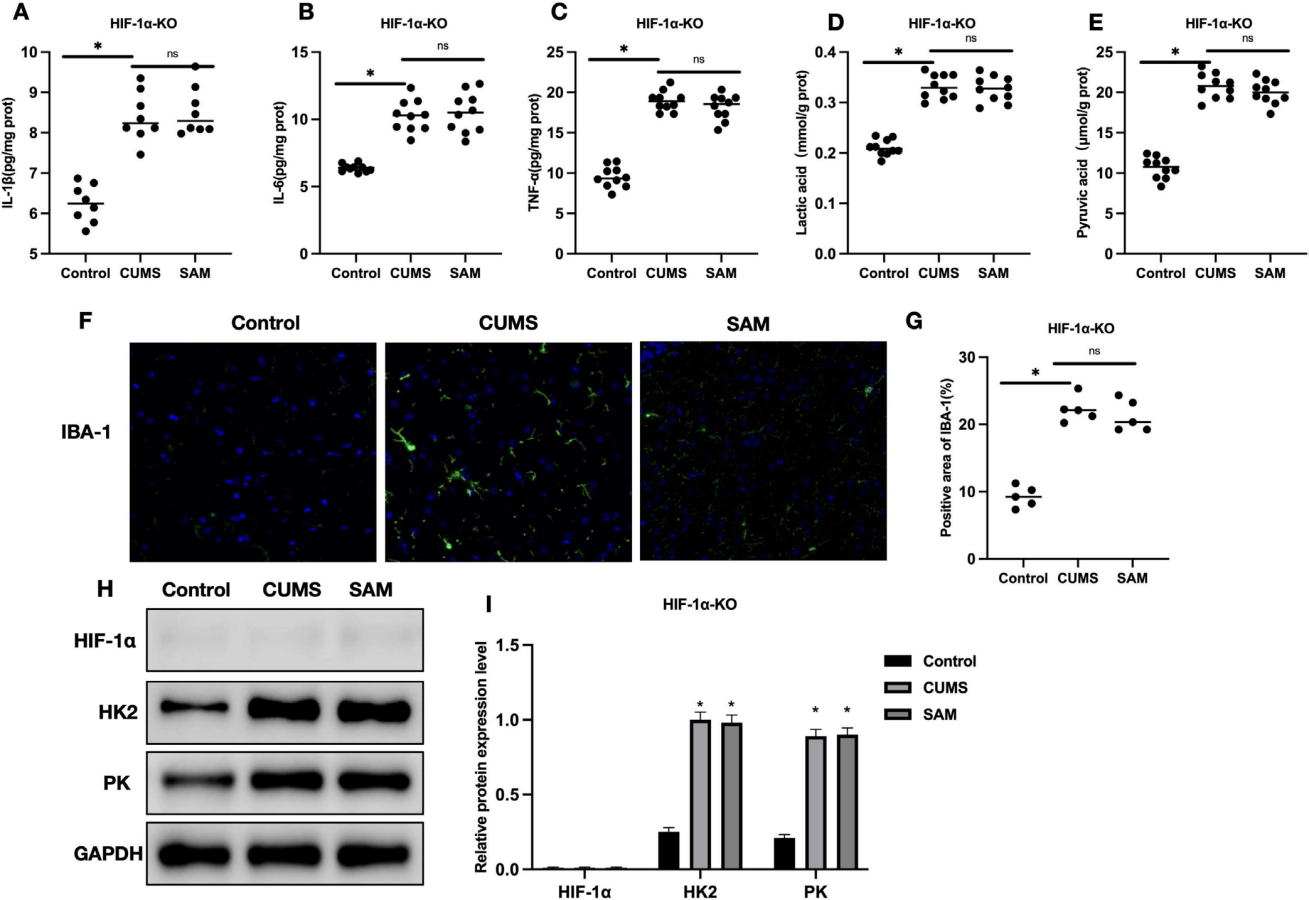
The effect of SAM on pathology in CUMS mice after HIF‐1α‐KO. (A–C) ELISA (*n* = 10); no significant difference in the levels of IL‐1β, IL‐6, and TNF‐α in brain tissue between CUMS and SAM groups. (D, E) Lactate and pyruvate detection (*n* = 10); no significant difference in the levels of pyruvate and lactate between CUMS and SAM groups. (F, G) IBA‐1 staining (*n* = 5); no significant difference in the number of IBA‐1 positive cells between CUMS and SAM groups. (H, I) Relative protein expression levels (*n* = 5); HIF‐1α was not significantly expressed due to the effect of shRNA, and there was no significant difference in the levels of HK2 and PK between CUMS and SAM groups. Group comparison, *p < 0.05; ns, *p* > 0.05.

Inflammatory factor detection showed no significant difference in the levels of IL‐1β, IL‐6, and TNF‐α in brain tissue between CUMS and SAM groups (Figure 5A–C). Lactate and pyruvate detection also showed no significant difference in the levels of pyruvate and lactate between CUMS and SAM groups (Figure 5D,E). IBA‐1 staining showed no significant difference in the number of IBA‐1 positive cells between CUMS and SAM groups (Figure 5F,G). Relative protein expression levels showed that HIF‐1α was not significantly expressed due to the effect of shRNA, and there was no significant difference in the levels of HK2 and PK between CUMS and SAM groups (Figure 5H,I).

## 4 Molecular Docking and Dynamics Analysis of SAM and HIF‐1α

**FIGURE 6:**
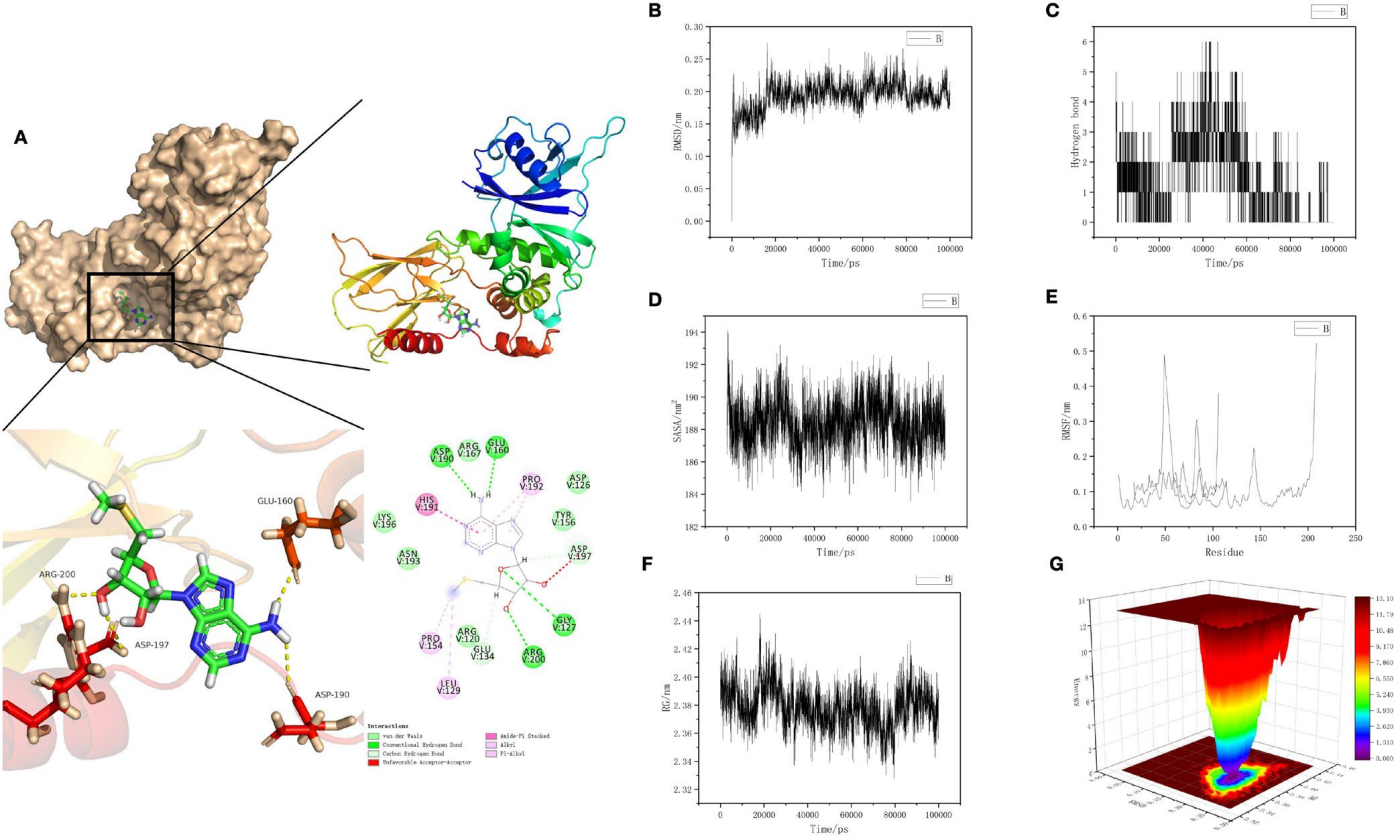
Molecular docking and dynamics analysis of SAM and HIF‐1α. (A) Molecular docking: SAM formed hydrogen bonds with ARG, GLY, ASP, and GLU, and had molecular bonds with HIS, PRO, and LEU. SAM had binding relationships in the hydrophobic pocket of HIF‐1α. (B–F) Dynamics analysis results: The RMSD was stable between 0.10 and 0.25, indicating stable molecular binding. The SASA showed stable conformations at 186–192, and the RMSF results showed high fitting. (G) Energy trap analysis results.

After molecular docking of SAM and HIF‐1α, SAM formed hydrogen bonds with ARG, GLY, ASP, and GLU, and had molecular bonds with HIS, PRO, and LEU. SAM had binding relationships in the hydrophobic pocket of HIF‐1α (Figure 6A). The dynamics analysis results showed that the RMSD was stable between 0.10 and 0.25, indicating stable molecular binding. The SASA showed stable conformations at 186–192, and the RMSF results showed high fitting (Figure 6B–F). We performed energy analysis on the binding, and the energy trap is shown in the Figure 6G.

## 5 Discussion

Microglia are considered the main innate immune cells in the central nervous system and are key cellular regulators in the occurrence and development of neuroinflammation [17, 18]. In the normal central nervous system, microglia are in a resting state, with supportive, nutritional, and immune surveillance functions [19]. However, in pathological conditions such as neuronal damage and infection, microglia are easily activated into amoeboid phagocytes, producing and releasing a large number of pro‐inflammatory factors to phagocytize invading microorganisms, clear damaged and dead cells, and restore and maintain the homeostasis of the central nervous system [20]. Among the activation types of microglia, M1‐type microglia release large amounts of inflammatory mediators such as IL‐6, IL‐1β, and TNF‐α when activated [21, 22, 23], which have neurotoxic effects on neurons, leading to neuronal degeneration and death [24]. The activation of microglia and the induced inflammatory response are prone to induce neuronal damage [25].

In patients with depression, microglia are abnormally activated, and the release of pro‐inflammatory factors in the cerebrospinal fluid is increased [25, 26]. This indicates that microglia‐mediated neuroinflammation plays an important role in the occurrence and development of depression. Inhibiting microglia activation‐mediated inflammatory response can be a potential therapeutic target for depression [27]. When macrophages are stimulated by LPS and activated into M1 type, the cell sugar metabolism shifts from oxidative phosphorylation to aerobic glycolysis [28], characterized by increased glucose uptake, increased lactate release, enhanced pentose phosphate pathway, decreased NAD+ content, and decreased mitochondrial oxygen consumption, similar to the Warburg effect in tumor cells [29, 30]. Compared with mitochondrial oxidative phosphorylation, although glycolysis is less efficient in utilizing glucose, it produces more energy per unit time to meet the rapidly increased ATP demand of macrophages due to biosynthesis [31]. After M1‐type activation of macrophages, the expression of glucose transporters, hexokinase, 2,6‐diphosphofructose kinase, pyruvate dehydrogenase kinase, and lactate dehydrogenase is upregulated, thereby promoting glycolysis and inhibiting the activity of pyruvate dehydrogenase to prevent the conversion of pyruvate to acetyl‐CoA [32], ultimately inhibiting the tricarboxylic acid cycle. Inflammatory reactions cause an increase in the expression of hypoxia‐inducible factor (HIF‐1α), which is considered a key molecule in the shift from oxidative phosphorylation to aerobic glycolysis in inflammatory cells [33]. LPS induces an increase in the accumulation of succinate, a tricarboxylic acid cycle intermediate, in macrophages, thereby increasing the stability of HIF‐1α protein, ultimately leading to enhanced glycolysis and increased expression of inflammatory factors [34]. Another study found that LPS‐activated PI3K/Akt/HIF‐1α signaling may be a key mechanism for the shift of dendritic cells to aerobic glycolysis, and it was also found that AMPK signaling is involved in the activation of dendritic cells and aerobic glycolysis [35].

SAM is a nucleoside small molecule in deer antler with important activity. Previous research on the antineurological disease effects of deer antler has focused on deer antler peptides and polysaccharides, neglecting SAM. We extracted SAM from deer antler and found that SAM has a significant effect on improving depression‐like behaviors in CUMS mice. Microglia‐mediated inflammatory responses are one of the main factors in the progression of depression. SAM can inhibit neuroinflammation in CUMS mice, improve mouse behavior, significantly improve mouse cognition and mobility, and reduce the levels of pyruvate and lactate. Pyruvate and lactate are intermediate metabolites of glycolysis. SAM also inhibits the action of glycolytic enzymes, reduces the expression of HK2 and PK, and inhibits the levels of HIF‐1α. HIF‐1α‐mediated glycolysis is the main signal for microglial inflammatory responses. When we used shRNA to silence HIF‐1α, the results showed that the effect of SAM was inhibited. SAM could not further improve CUMS behavior and neuroinflammation, and glycolysis levels were not further inhibited. We believe that SAM targets HIF‐1α to exert its effects. When HIF‐1α is lowly expressed, SAM loses its target, and thus its effect is inhibited.

## 6 Conclusion

We found that the active ingredient S‐adenosylmethionine in deer antler has a good therapeutic effect on depression. SAM can exert its effects by inhibiting neuroinflammation through regulating glycolysis in microglia, and its mechanism is related to the inhibition of HIF‐1α. SAM affects glycolysis through HIF‐1α to exert its effects. Our study provides new references for the pharmacological effects of deer antler. However, we have not yet touched the role of SAM in clinical practice, which is also the direction of future research.

## Ethics Statement

The study was approved by the Ethics Committee.

## Consent

All authors approval published the article.

## Conflicts of Interest

The authors declare no conflicts of interest.

## Data Availability

Data sharing not applicable to this article as no datasets were generated or analysed during the current study.

## References

[1] Y. Wu , Y. Zhu , S. Zheng , and D. Mingxing , “Resveratrol Alleviates Depressive‐Like Behavior via the Activation of SIRT1/NF‐κB Signaling Pathway in Microglia,” Future Science OA 11, no. 1 (2025): 2463852. 39967065 10.1080/20565623.2025.2463852 PMC11845112

[2] L. Zhai , H. Shen , S. Wu , et al., “Deer Antler Polypeptides Inhibit Microglial Activation via TREM2 to Improve Behavior and Neuroinflammation in CUMS Mice,” International Immunopharmacology 150 (2025): 114284. 39955919 10.1016/j.intimp.2025.114284

[3] X. Zhang , Q. Liu , S. Li , et al., “Traditional Pediatric Massage Exerted an Antidepressant Effect and Activated IGF‐1/Nrf2 Pathway in CUMS‐Exposed Adolescent Rats,” Journal of Neuroimmunology 400 (2025): 578554. 39954614 10.1016/j.jneuroim.2025.578554

[4] H. Wang , J. Peng , H. Zhu , et al., “Microglia Stimulation Produces Antidepressant‐Like Effects in a Mouse Depression Model Induced by Adolescent Chronic Unpredictable Stress,” Physiology & Behavior 291 (2025): 114782. 39672484 10.1016/j.physbeh.2024.114782

[5] X. Lei , D. Zhao , T. Chen , et al., “Exploring the Active Components and Potential Mechanisms of Zhimu‐Huangbai Herb‐Pair in the Treatment of Depression,” Phytomedicine 138 (2025): 156365. 39904199 10.1016/j.phymed.2025.156365

[6] Y. Wu , L. Yang , W. Jiang , X. Zhang , and Z. Yao , “Glycolytic Dysregulation in Alzheimer's Disease: Unveiling New Avenues for Understanding Pathogenesis and Improving Therapy,” Neural Regeneration Research 20, no. 8 (2025): 2264–2278. 39101629 10.4103/NRR.NRR-D-24-00190 PMC11759019

[7] C. W. Chang , A. Bale , R. Bhargava , and B. A. C. Harley , “Glioblastoma Drives Protease‐Independent Extracellular Matrix Invasion of Microglia,” Materials Today Bio 31 (2025): 101475. 10.1016/j.mtbio.2025.101475 PMC11787038 39896278

[8] X. Xia , W. Chen , T. Zhou , et al., “TEPP‐46 Inhibits Glycolysis to Promote M2 Polarization of Microglia After Ischemic Stroke,” International Immunopharmacology 149 (2025): 114148. 39904037 10.1016/j.intimp.2025.114148

[9] L. Li , L. Wang , W. Ding , et al., “The Improvement Effects of Sika Deer Antler Protein in an Alzheimer's Disease Mouse Model via the Microbe‐Gut‐Brain Axis,” Food Science & Nutrition 13, no. 1 (2024): e4656. 39803278 10.1002/fsn3.4656 PMC11717054

[10] B. Yao , M. Zhang , X. Leng , and D. Zhao , “Proteomic Analysis of the Effects of Antler Extract on Chondrocyte Proliferation, Differentiation and Apoptosis,” Molecular Biology Reports 46, no. 2 (2019): 1635–1648. 30680597 10.1007/s11033-019-04612-1

[11] H. Li , Y. Xiang , Z. Zhu , et al., “Rifaximin‐Mediated Gut Microbiota Regulation Modulates the Function of Microglia and Protects Against CUMS‐Induced Depression‐Like Behaviors in Adolescent Rat,” Journal of Neuroinflammation 18, no. 1 (2021): 254. 34736493 10.1186/s12974-021-02303-y PMC8567657

[12] X. M. Zhou , C. Y. Liu , Y. Y. Liu , et al., “Xiaoyaosan Alleviates Hippocampal Glutamate‐Induced Toxicity in the CUMS Rats via NR2B and PI3K/Akt Signaling Pathway,” Frontiers in Pharmacology 12 (2021): 586788. 33912031 10.3389/fphar.2021.586788 PMC8075411

[13] R. Liu , H. Zhou , H. Qu , et al., “Effects of Aerobic Exercise on Depression‐Like Behavior and TLR4/NLRP3 Pathway in Hippocampus CA1 Region of CUMS‐Depressed Mice,” Journal of Affective Disorders 341 (2023): 248–255. 37634821 10.1016/j.jad.2023.08.078

[14] W. Tao , J. Ruan , R. Wu , et al., “A Natural Carotenoid Crocin Exerts Antidepressant Action by Promoting Adult Hippocampal Neurogenesis Through Wnt/β‐Catenin Signaling,” Journal of Advanced Research 43 (2023): 219–231. 36585110 10.1016/j.jare.2022.02.015 PMC9811320

[15] R. Lu , L. Zhang , H. Wang , M. Li , W. Feng , and X. Zheng , “Echinacoside Exerts Antidepressant‐Like Effects Through Enhancing BDNF‐CREB Pathway and Inhibiting Neuroinflammation via Regulating Microglia M1/M2 Polarization and JAK1/STAT3 Pathway,” Frontiers in Pharmacology 13 (2023): 993483. 36686689 10.3389/fphar.2022.993483 PMC9846169

[16] R. Troubat , P. Barone , S. Leman , et al., “Neuroinflammation and Depression: A Review,” European Journal of Neuroscience 53, no. 1 (2021): 151–171. 32150310 10.1111/ejn.14720

[17] K. Nagamotocombs , R. J. Morecraft , W. G. Darling , W. G. Darling , and C. K. Combs , “Long‐Term Gliosis and Molecular Changes in the Cervical Spinal Cord of the Rhesus Monkey After Traumatic Brain Injury,” Journal of Neurotrauma 27, no. 3 (2010): 565–585. 20030560 10.1089/neu.2009.0966 PMC2867631

[18] G. W. Kreutzberg , “Microglia: Intrinsic Immuneffector Cell of the Brain,” Brain Research. Brain Research Reviews 20, no. 3 (1995): 269–287. 7550361 10.1016/0165-0173(94)00015-h

[19] A. J. Brucekeller , “Microglial‐Neuronal Interactions in Synaptic Damage and Recovery,” Journal of Neuroscience Research 58, no. 1 (1999): 191–201. 10491582 10.1002/(sici)1097-4547(19991001)58:1<191::aid-jnr17>3.0.co;2-e

[20] M. È. Tremblay , R. L. Lowery , and A. K. Majewska , “Microglial Interactions With Synapses Are Modulated by Visual Experience,” PLoS Biology 8, no. 11 (2010): e1000527. 21072242 10.1371/journal.pbio.1000527 PMC2970556

[21] M. Olah , K. Biber , J. Vinet , and H. W. Boddeke , “Microglia Phenotype Diversity J,” CNS & Neurological Disorders Drug Targets 10, no. 1 (2011): 108–118. 21143141 10.2174/187152711794488575

[22] E. L. Pearce and E. J. Pearce , “Metabolic Pathways in Immune Cell Activation and Quiescence,” Immunity 38, no. 4 (2013): 633–643. 23601682 10.1016/j.immuni.2013.04.005 PMC3654249

[23] T. Pfeiffer , S. Schuster , and S. Bonhoeffer , “Cooperation and Competition in the Evolution of ATP‐Producing Pathways,” Science 292, no. 5516 (2001): 504–507. 11283355 10.1126/science.1058079

[24] G. M. Tannahill , A. M. Curtis , J. Adamik , et al., “Succinate Is an Inflammatory Signal That Induces IL‐1β Through HIF‐1α,” Nature 496, no. 7444 (2013): 238–242. 23535595 10.1038/nature11986 PMC4031686

[25] B. Beltrán , A. Mathur , M. R. Duchen , J. D. Erusalimsky , and S. Moncada , “The Effect of Nitric Oxide on Cell Respiration: A Key to Understanding Its Role in Cell Survival or Death,” Proceedings of the National Academy of Sciences of the United States of America 97 (2000): 14602–14607. 11121062 10.1073/pnas.97.26.14602 PMC18965

[26] B. Kelly , G. M. Tannahill , M. P. Murphy , and L. A. O'Neill , “Metformin Inhibits the Production of Reactive Oxygen Species From NADH:Ubiquinone Oxidoreductase to Limit Induction of Interleukin‐1β (IL‐1β) and Boosts Interleukin‐10 (IL‐10) in Lipopolysaccharide (LPS)‐Activated Macrophages,” Journal of Biological Chemistry 290, no. 33 (2015): 20348–20359. 26152715 10.1074/jbc.M115.662114 PMC4536441

[27] S. E. Weinberg , L. A. Sena , and N. S. Chandel , “Mitochondria in the Regulation of Innate and Adaptive Immunity,” Immunity 42, no. 3 (2015): 406–417. 25786173 10.1016/j.immuni.2015.02.002 PMC4365295

[28] A. D. D'Souza , N. Parikh , S. M. Kaech , and G. S. Shadel , “Convergence of Multiple Signaling Pathways Is Required to Coordinately Up‐Regulate mtDNA and Mitochondrial Biogenesis During T Cell Activation,” Mitochondrion 7, no. 6 (2007): 374–385. 17890163 10.1016/j.mito.2007.08.001 PMC2692272

[29] S. K. Biswas and A. Mantovani , “Orchestration of Metabolism by Macrophages,” Cell Metabolism 15, no. 4 (2012): 432–437. 22482726 10.1016/j.cmet.2011.11.013

[30] S. Galvánpeña and L. A. J. O'Neill , “Metabolic Reprograming in Macrophage Polarization,” Frontiers in Immunology 5 (2014): 420. 25228902 10.3389/fimmu.2014.00420 PMC4151090

[31] J. I. Odegaard and A. Chawla , “Alternative Macrophage Activation and Metabolism,” Annual Review of Pathology 6, no. 1 (2011): 275–297. 10.1146/annurev-pathol-011110-130138 PMC3381938 21034223

[32] L. A. Sena , S. Li , A. Jairaman , et al., “Mitochondria Are Required for Antigen‐Specific T Cell Activation Through Reactive Oxygen Species Signaling,” Immunity 38, no. 2 (2013): 225–236. 23415911 10.1016/j.immuni.2012.10.020 PMC3582741

[33] M. Sukumar , J. Liu , G. U. Mehta , et al., “Mitochondrial Membrane Potential Identifies Cells With Enhanced Stemness for Cellular Therapy,” Cell Metabolism 23, no. 1 (2016): 63–76. 26674251 10.1016/j.cmet.2015.11.002 PMC4747432

[34] M. M. Kamiński , S. W. Sauer , M. Kamiński , et al., “T Cell Activation Is Driven by an ADP‐Dependent Glucokinase Linking Enhanced Glycolysis With Mitochondrial Reactive Oxygen Species Generation,” Cell Reports 2, no. 5 (2012): 1300–1315. 23168256 10.1016/j.celrep.2012.10.009

[35] D. K. Singh , D. Kumar , Z. Siddiqui , S. K. Basu , V. Kumar , and K. V. Rao , “The Strength of Receptor Signaling Is Centrally Controlled Through a Cooperative Loop Between Ca2+ and an Oxidant Signal,” Cell 121, no. 2 (2005): 281–293. 15851034 10.1016/j.cell.2005.02.036

